# SOX11 Is Regulated by EGFR-STAT3 and Promotes Epithelial–Mesenchymal Transition in Head and Neck Squamous Cell Carcinoma

**DOI:** 10.3390/cells15010084

**Published:** 2026-01-04

**Authors:** Jiayi Peng, Li Cui, Mian Guo, Yi Liu, Wanqi Jia, Kaori Misuno, Jeremy Barrett, Diana Messadi, Shun-Fa Yang, Shen Hu

**Affiliations:** 1 Division of Oral and Systemic Health Sciences, School of Dentistry, University of California, Los Angeles, CA 90095, USA; 2 Jonsson Comprehensive Cancer Center, University of California, Los Angeles, CA 90095, USA; 3 School of Biological Sciences, University of California, Irvine, CA 92697, USA; wanqij2@uci.edu; 4 Institute of Medicine, Chung Shan Medical University, Taichung 40201, Taiwan; 5 Department of Medical Research, Chung Shan Medical University Hospital, Taichung 40201, Taiwan; 6 California NanoSystems Institute, University of California, Los Angeles, CA 90095, USA

**Keywords:** head and neck squamous cell carcinoma, SOX11, STAT3, epithelial–mesenchymal transition

## Abstract

The transcription factor SOX11 is implicated in tumor progression across multiple types of cancers, including head and neck squamous cell carcinoma (HNSCC). However, its mechanistic role in HNSCC remains elusive. In this study, we found that the expression of SOX11 was induced by epidermal growth factor (EGF) but suppressed by an epidermal growth factor receptor (EGFR) inhibitor in HNSCC cells. The signal transducer and activator of transcription 3 (STAT3) bound to the Sox11 gene promoter and transcriptionally upregulated the expression of Sox11 in HNSCC cells. Meanwhile, analyses of The Cancer Genome Atlas (TCGA) gene expression datasets indicated that Sox11 gene expression was significantly overexpressed in HNSCC versus adjacent normal tissues and correlated with those of most epithelial–mesenchymal transition transcription factors (EMT-TFs) and marker genes. Knockdown of SOX11 significantly downregulated the expression of EMT-related genes, including EMT-TFs, vimentin, fibronectin, and N-cadherin, but significantly upregulated E-cadherin and vice versa when SOX11 was overexpressed. Collectively, our studies demonstrated that SOX11 was regulated by EGF-EGFR-STAT3 signals, promoting EMT in HNSCC.

## 1. Introduction

Head and neck squamous cell carcinomas (HNSCCs), primarily originating from mucosal cells in the oral cavity, pharynx, and larynx, represent the sixth most prevalent cancer globally. In 2018 alone, there were approximately 890,000 new cases and 450,000 deaths worldwide. Alarmingly, the incidence of HNSCC is projected to increase by 30% by 2030 [1,2]. Current therapeutic strategies, including surgery, radiotherapy, chemotherapy, and immunotherapy, are frequently compromised by therapy resistance, leading to locoregional recurrence or distant metastasis in over 50% of patients [3]. This underscores the critical need to elucidate the molecular drivers of HNSCC progression and identify novel therapeutic targets for improved treatment of the devastating disease.

The SOX transcription factor family, defined by a conserved high-mobility group (HMG) DNA-binding domain and originating from the discovery of the SRY (sex-determining region Y) protein, comprises 11 subgroups (SoxA–SoxK) based on structural and sequence homology [4,5,6,7]. SOX proteins are pivotal regulators of diverse developmental processes, including chondrogenesis and neurogenesis [8], and modulate adult tissue homeostasis by influencing cell survival, migration, regeneration, and apoptosis [9].

Among these, Sox11 belongs to the SoxC subgroup alongside Sox4 and Sox12. SOX11 regulates epithelial–mesenchymal interactions during organogenesis, such as in kidney and lung development [10]. Notably, SOX11 is overexpressed in a wide spectrum of human malignancies, including mantle cell lymphoma, glioma, breast and ovarian carcinomas, gastrointestinal cancers, HNSCC, leukemia, Burkitt’s lymphoma, and pediatric medulloblastoma and ependymoma [11,12,13,14,15,16,17,18,19,20,21,22,23,24,25,26,27,28,29,30]. While implicated in promoting proliferation and inhibiting apoptosis [31,32], the precise mechanistic role of SOX11 in tumorigenesis remains largely elusive. Therefore, this study aims to delineate the upstream regulators and downstream effectors of SOX11 in HNSCC, providing insights into its functional role in head and neck cancer progression.

## 2. Materials and Methods

### 2.1. Cell Culture

The UM1 and UM2 cell lines were kindly provided by Professor Yong Kim (University of California, Los Angeles, CA, USA), while UMSCC5 and UMSCC6 were obtained from the University of Michigan. All four HNSCC cell lines (UM1, UM2, UMSCC5, and UMSCC6) were maintained in Dulbecco’s modified Eagle’s medium (DMEM) supplemented with 10% fetal bovine serum, 100 U/mL penicillin G, and 100 μg/mL streptomycin (Invitrogen, Carlsbad, CA, USA). Cells were cultured at 37 °C in a humidified incubator containing 5% CO_2_ and 95% air, and the medium was refreshed every 2–3 days.

### 2.2. Real-Time Quantitative PCR (qPCR)

The RNeasy Mini Kit (Qiagen, Valencia, CA, USA) was used to isolate total RNA from the cultured cells, and the SuperScript III Reverse Transcriptase (Invitrogen) was used to perform the synthesis of first-strand cDNA. Afterwards, the CFX96 Real-Time PCR system (Bio-Rad, Hercules, CA, USA) and Light Cycler^®^ 480 SYBR Green I MasterMix (Roche, Indianapolis, IN, USA) were used to amplify the cDNA. The PCR program consisted of an initial denaturation at 95 °C for 3 min, followed by 40 cycles of 95 °C for 10 s and 60 °C for 30 s. Primers used for qPCR analyses are provided in Supplementary Table S1. Gene expression was quantified in triplicate, and β-actin served as the internal control. Relative expression levels were calculated using the 2^−ΔΔCT^ method, where ΔCT = CT(target) − CT(β-actin).

### 2.3. Western Blotting

SDS-PAGE was used to separate equal amounts of total proteins from each sample, and the proteins were transferred onto a nitrocellulose membrane with the Trans-blot SD semi-dry transfer cell (Bio-Rad, Hercules, CA, USA). Blocking of the membrane was carried out with 5% non-fat dry milk (Santa Cruz Biotech, Dallas, TX, USA) for one hour. Then, the membrane was incubated with anti-SOX11 (Santa Cruz Biotech, #Sc-20,096), anti-TWIST1 (Proteintech), or anti-GAPDH (GeneTex, #GTX100118) primary antibody, followed by incubation with an HRP-conjugated secondary antibody (GE Healthcare, Piscataway, NJ, USA). ECL-Plus Kit (GE Healthcare) was used for signal detection, and NIH Image J (V1.41) was used for protein quantification.

### 2.4. Chromatin Immunoprecipitation (ChIP)

Chromatin immunoprecipitation experiments were conducted in UM1 and UMSCC5 cells using a commercially available ChIP assay kit (Millipore, Billerica, MA, USA; #17–295). Cells were initially rinsed with PBS and incubated with 1% formaldehyde for 10 min at room temperature to stabilize protein–DNA interactions, after which the reaction was terminated by the addition of glycine to a final concentration of 125 mM. Cells were subsequently washed twice with ice-cold PBS and lysed in a RIPA buffer (150 mM NaCl, 1% Igepal CA-630, 0.5% deoxycholate, 0.1% SDS, 50 mM Tris-HCl, pH 8). Chromatin was fragmented by sonication to an average size of 200–1000 bp.

To perform ChIP, 30 μL of Protein A agarose/salmon sperm DNA (50% Slurry) was utilized to pre-clear 1 mg of protein extract for 30 min. After removal of the pellet by brief centrifugation, the supernatants were incubated overnight at 4 °C with antibodies against SOX11 (Santa Cruz Biotechnology, Dallas, TX, USA; #Sc-20,096), STAT3 (Proteintech, Rosemont, IL, USA), or phosphorylated STAT3 (Life Technologies, Carlsbad, CA, USA; MA5-41192). Immune complexes were then captured by incubation with Protein A agarose/salmon sperm DNA beads for an additional 1 h at 4 °C. Following the collection of the pellet, sequential washes were performed using a low-salt Immune Complex Wash Buffer, high-salt Immune Complex Wash Buffer, LiCl Immune Complex Wash Buffer, and the TE buffer. The immunocomplex was eluted using 1% SDS/0.1 M NaHCO_3_ at 65 °C, and protein–DNA crosslinks were reversed by incubation with 200 mM NaCl for 4 h at 65 °C. Purified DNA was recovered using the DNA Clean & Concentrator kit (Zymo Research, Irvine, CA, USA; #D4003) and analyzed by qPCR with primers specific for the Sox11 and Twist1 promoter regions (Supplementary Table S1).

### 2.5. Luciferase Reporter Assay

Luciferase-based transcriptional activity assays were used to assess whether STAT3 regulates Sox11 promoter activity in HNSCC cells. UM1 and UMSCC5 cells were plated in 24-well culture plates and, at approximately 80% confluence, transfected with empty promoter reporter vector (pLightSwitch_Prom, #S790005, 200 ng, SwitchGear Genomics, Carlsbad, CA, USA), Sox11 gene promoter reporter vector (Sox11-Prom, SwitchGear Genomics), Sox11-Prom with Stat3 plasmid (STAT3), or Sox11-Prom with mutated Stat3 plasmid (STAT3∆DBD). Transfections were carried out using Lipofectamine 2000 (Invitrogen, Carlsbad, CA, USA) according to the manufacturer’s recommendations.

In parallel, the ability of SOX11 to activate the Twist1 promoter was examined using a similar experimental approach. UM1 and UMSCC5 cells were transfected with empty promoter reporter vector (pLightSwitch_Prom), Twist1 gene promoter reporter vector (Twist-Prom, SwitchGear Genomics), Twist1-Prom with wild-type Sox11 plasmid, or Twist1-Prom with mutated Sox11 (Sox11FΔTAD) plasmid. Twenty-four hours after transfection, luciferase activity was quantified using a commercial reporter assay system (SwitchGear Genomics). Each experiment was performed in triplicate.

### 2.6. Capillary Electrophoresis-Based Mobility Shift Assay (CEMSA)

CEMSA was performed on a CE system (Moorpark Instruments, Brea, CA, USA) with a laser-induced fluorescence (LIF) detector (488 nm excitation and 520 nm emission). Separation was performed at −10 kV using a neutral-coated capillary (Beckman Coulter, 50 μm I.D., 30 cm length, 20 cm effective length) with a running buffer of 40 mM Tris–borate and 1.0 mM EDTA (pH 8.0). Samples were injected at −10 kV for 3 s. Before each run, the capillary was conditioned by washing with a running buffer for 2 min. Buffers and running solutions were filtered through 0.2 mm pore-size filters.

Single-stranded oligonucleotides (SSOs) were obtained from Integrated DNA Technologies (San Diego, CA, USA). Binding experiments between STAT3 and the predicted promoter DNA sequence were conducted and then evaluated by CEMSA. Forward oligonucleotides (GTGCCGGGAAG) were labelled at their 5′ ends with 6-carboxyfluorescein (6-FAM). Equimolar forward and reverse oligonucleotides were then mixed and annealed at 95 °C according to the procedure reported in a previous study [33]. STAT3 at a concentration of 2 μM was added to 6-FAM-labelled DNAs in binding buffer (10 mM Tris–HCl, pH 8.0, 3 mM MgCl_2_, 50 mM NaCl, 0.1 mM EDTA, 0.1% NP-40, 2 mM DTT, 5% glycerol and 0.4 mg/mL BSA) and incubated at 37 C for 15 min [33] prior to CE-LIF analysis.

### 2.7. Gene Silencing with siRNA

For gene silencing studies, UM1 and UMSCC5 cells were seeded into 6-well plates and subjected to transient siRNA-mediated knockdown. Transfections were performed using an RNAiMAX reagent in accordance with the manufacturer’s protocol. Two independent, pre-validated siRNAs targeting SOX11, along with a non-targeting siRNA control (Sigma, St. Louis, MO, USA), were introduced into the cultures following complex formation with the transfection reagent. Twenty-four hours after transfection, siRNA-containing medium was replaced with fresh complete growth medium, and the cells were maintained for downstream analyses. The reduction in SOX11 protein levels was verified by Western blot analysis.

### 2.8. Plasmids and Gene Overexpression

Plasmid-based gene overexpression in HNSCC cells was carried out using Lipofectamine 2000 as the transfection reagent. Expression vectors encoding full-length SOX11 (pCMV-Sox11F) and a transactivation domain-deficient mutant (pCMV-Sox11FΔTAD) were kindly provided by Professor Angie Rizzino [34] and Professor Kathryn Albers [35]. The Sox11F and Sox11FΔTAD constructs were generated using primers Sox-11F (5′-CGTGCTGGTACCGCCACCATGGACTACAAGGACGACGATGATATGGTGCAGCAGGCCGAGAGC-3′) and Sox11TAD (5′-CTCTACTACAGCTTCAAGTGAGCGGCCGCAAACATCACCAAGCAGCAG-3′), respectively, as previously described [17].

### 2.9. Xenograft Mouse Model

The mouse model studies were approved by the Animal Research Committee at the University of California, Los Angeles. The 4- to 5-week-old NSG male mice were purchased from the Jackson Laboratory (Bar Harbor, ME, USA). The cell suspension mixture was made by mixing the HNSCC cells with Matrigel (BD Biosciences) at a 1:1 ratio. Then, a 100 μL cell suspension (2 × 10^6^ cells) was injected into the dorsal flank of each mouse subcutaneously. After 4 weeks, the mice were euthanized, and the tumors were collected. The tumor size and volume are measured, followed by immunohistochemistry (IHC) analysis on tumor tissues. To perform the IHC, the FFPE sections were treated with 0.3% H_2_O_2_ in methanol after washing with xylene, 100% ethanol, 95% ethanol, and 80% ethanol, and PBS. After blocking with 5% BSA, the slides were incubated with primary antibodies, including anti-Ki67 (32270-1-AP), anti-PCNA (10205-2-AP), or anti-TWIST1 (25465-1-AP) (ProteinTech, Rosemont, IL 60018, USA), and subsequently with an HRP-linked secondary antibody.

### 2.10. Correlation Gene Expression Analysis of Sox11 with EMT-Related Genes in HNSCC

The gene expression data of HNSCC tissues (n = 522, mainly oral cavity, larynx, oropharynx, and hypopharynx) and normal tissues (n = 44) were obtained from the TCGA database (v37.0) of the National Cancer Institute Genomic Data Commons (https://gdc.cancer.gov/). To analyze the gene expression correlation between Sox11 and EMT transcription factors (EMT-TFs), vimentin, fibronectin, N-cadherin, and E-cadherin, the mRNA expression levels were log2-transformed, and R-studio (version 4.1) was used to generate the Pearson correlation graphs.

### 2.11. Statistical Analysis

Student’s *t*-test and one-way ANOVA (MedCalc, Ostend, Belgium) were used to perform statistical analysis. The data were expressed as the mean ± standard deviation. A *p*-value smaller than 0.05 was considered statistically significant for all data analyses.

## 3. Results

### 3.1. EGF Upregulates the Expression of SOX11 in HNSCC Cells

**Figure 1:**
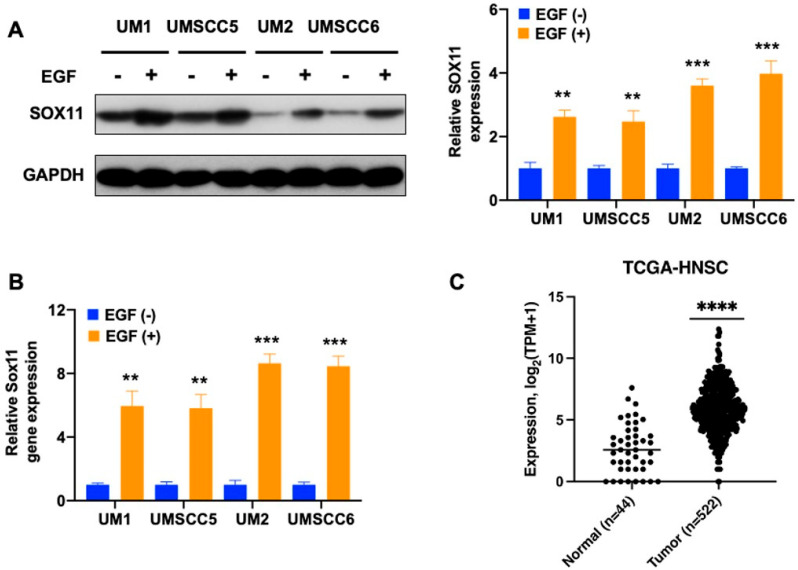
EGF induced the expression of SOX11 in HNSCC cells. (**A**) Western blot analysis of SOX11 expression in four HNSCC cell lines, UM1, UM2, UMSCC5, and UMSCC6, with or without EGF treatment. (**B**) qPCR analysis of Sox11 gene expression in UM1, UM2, UMSCC5, and UMSCC6 cell lines with or without EGF treatment. (**C**) Significant overexpression of Sox11 gene expression in HNSCC tissues (n = 522) versus adjacent normal tissues (n = 44) based on The Cancer Genome Atlas (TCGA) gene expression datasets. **, *p* < 0.01; ***, *p* < 0.001; ****, *p* < 0.0001.

Figure 1 shows the Western blot analysis of SOX11 protein levels (Figure 1A) and qPCR analysis of Sox11 mRNA expression levels (Figure 1B) between EGF-treated and untreated HNSCC cells. The results indicate that the EGF supplement significantly upregulates both the protein and mRNA expression of the Sox11 gene. The same results were consistently observed among all four HNSCC cell lines: UM1, UM2, UMSCC5, and UMSCC6. Furthermore, in Figure 1C, Sox11 gene expression was significantly overexpressed in HNSCC tissues (n = 522) compared to adjacent normal tissues (n = 44) based on the TCGA gene expression datasets.

### 3.2. EGFR Inhibitor Suppresses the Expression of SOX11 in HNSCC Cells

**Figure 2:**
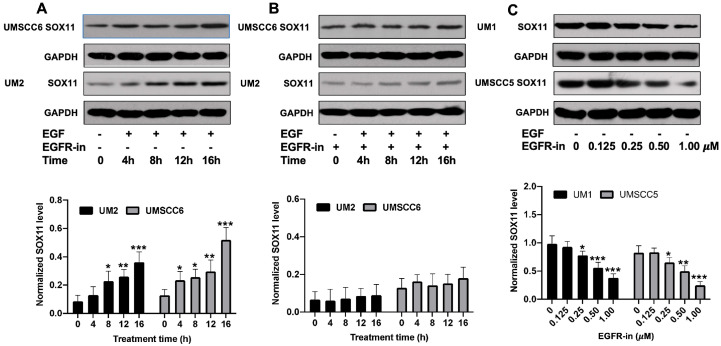
Effect of EGF and EGFR inhibitor on SOX11 expression in HNSCC cells. (**A**) Western blot analysis of SOX11 in UM2 and UMSCC6 cancer cells after treatment with only EGF at different time points: 0 h, 4 h, 8 h, 12 h, and 16 h. (**B**) Western blot analysis of SOX11 in UM2 and UMSCC6 cancer cells treated with EGF and EGFR inhibitor (N-(3-ethynylphenyl)-6,7-bis(2-methoxyethoxy)-4-quinazolinamine) at different time points: 0 h, 4 h, 8 h, 12 h, and 16 h. (**C**) Western blot analysis of SOX11 in UM1 and UMSCC5 cancer cells after treatment with the indicated dose of the EGFR inhibitor: 0 µM, 0.125 µM, 0.25 µM, 0.50 µM, and 1.00 µM. *, *p* < 0.05; **, *p* < 0.01; ***, *p* < 0.001.

Figure 2 shows a time-dependent study of SOX11 expression in low-invasive UM2 and UMSCC6 cancer cells treated with EGF. Western blot analysis indicates that the SOX11 expression level in both UM2 and UMSCC6 cells significantly increased (Figure 2A) when N-(3-ethynylphenyl)-6,7-bis(2-methoxyethoxy)-4-quinazolinamine (EMEQA), an EGFR inhibitor, was absent. However, when EMEQA and EGF are both present in the culture medium, the expression of SOX11 was not significantly increased (Figure 2B). Figure 2C presents a dose-dependent study of the effect of EMEQA on the expression of SOX11 in highly invasive UM1 and UMSCC5 cells (Figure 2C). Higher doses of EMEQA led to a more significant decrease in SOX11 expression in HNSCC cells.

### 3.3. Sox11 Is Regulated by STAT3 in HNSCC Cells

**Figure 3:**
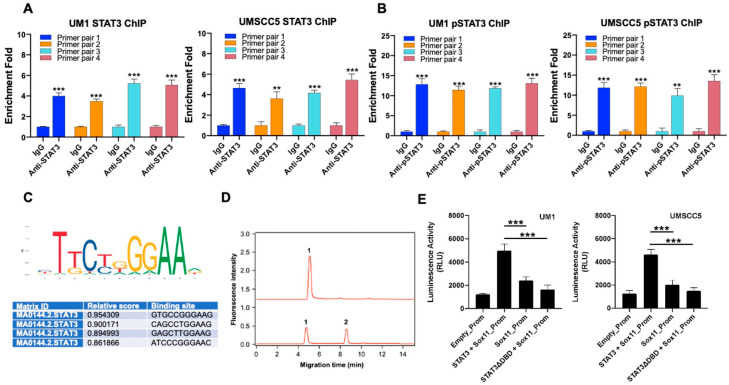
EGF-STAT3 signal upregulates SOX11 in HNSCC cancer cells. ChIP assays were performed with either STAT3 antibody (**A**) or phospho-STAT3 (pSTAT3) antibody (**B**). The immunoprecipitated DNA fragments were quantified with qPCR using 4 pairs of primers for the Sox11 gene promoter. Data were measured in triplicate. **, *p* < 0.01; ***, *p* < 0.001. (**C**) STAT3 consensus binding sites obtained from the JASPER database. JASPAR predicted GTGCCGGGAAG as the top-ranked binding sequence of STAT3 on the Sox11 gene promoter. (**D**) CEMSA of a binding mixture of the consensus binding sequence of the Sox11 gene promoter and STAT3. Neutral coated capillary: 50 µm I.D., 20 cm effective length; separation voltage: −15 kV; buffer, 40 mM Tris–borate, 0.95 mM EDTA, pH 8.0; detection: laser-induced fluorescence (LIF). (**E**) Luciferase reporter assays of UM1 or UMSCC5 cells transfected with an empty promoter reporter vector, Sox11 gene promoter reporter (Sox11_Prom), Sox11_Prom and Stat3 plasmid, or Sox11-Prom and mutated Stat3 plasmid (STAT3∆DBD). Data were measured in triplicate. **, *p* < 0.01; ***, *p* < 0.001.

ChIP assays were performed in UM1 and UMSCC5 cells using anti-STAT3 and anti-pSTAT3 antibodies to determine if STAT3 or pSTAT3 binds to the Sox11 gene promoter in HNSCC cells. To measure the DNA enrichment within the immunoprecipitated samples, four pairs of primers of the Sox11 gene promoter (primer pairs #1, #2, #3, and #4) were used in performing real-time qPCR. Analyses of the qPCR results were carried out by comparing data from anti-STAT3 or anti-pSTAT3 immunoprecipitated samples against the background signal of the negative control (IgG antibody) to calculate the enrichment fold. Both UM1 and UMSCC5 cells have significantly higher enrichment folds for the DNA fragments of the Sox11 gene promoter in the anti-STAT3-immunoprecipitated (Figure 3A) or anti-pSTAT3-immunoprecipitated (Figure 3B) samples than in the IgG-immunoprecipitated samples. More importantly, the enrichment folds for the DNA fragments of the Sox11 gene promoter are much higher for anti-pSTAT3-immunoprecipitated samples (Figure 3B) than anti-STAT3-immunoprecipitated samples (Figure 3A) in both cell lines (UM1 and UMSCC5).

The predicted transcription binding sites of STAT3 on the Sox11 gene promoter were obtained from the JASPAR database, and the binding site with the highest ranking was GTGCCGGGAAG (Figure 3C). As shown in Figure 3D (peak 2), capillary electrophoresis-based mobility shift assay (CEMSA) indicated that, due to STAT3’s binding to the predicted consensus DNA sequence, a second CE peak was formed.

Luciferase reporter assays were then performed on UM1 and UMSCC5 cells transfected with an empty promoter reporter plasmid, the Sox11 gene promoter reporter plasmid, the Sox11 gene promoter reporter with the Stat3 plasmid (STAT3), or the Sox11 gene promoter reporter and the mutated Stat3 plasmid (STAT3∆DBD) to further confirm that STAT3 regulates the expression of Sox11 in HNSCC cells. The results from luciferase assays of UM1 and UMSCC5 cells are shown in Figure 3E. As expected, the luciferase activities were significantly higher when the Sox11 gene promoter and STAT3 were co-transfected in UM1 or UMSCC5 cells than when the cells were transfected with the Sox11 gene promoter alone or with the Sox11 gene promoter reporter and mutated Stat3 plasmid (STAT3∆DBD).

### 3.4. SOX11 Upregulates EMT Transcription Factors (EMT-TFs), Vimentin, Fibronectin, and N-Cadherin While It Downregulates E-Cadherin in HNSCC Cells

**Figure 4:**
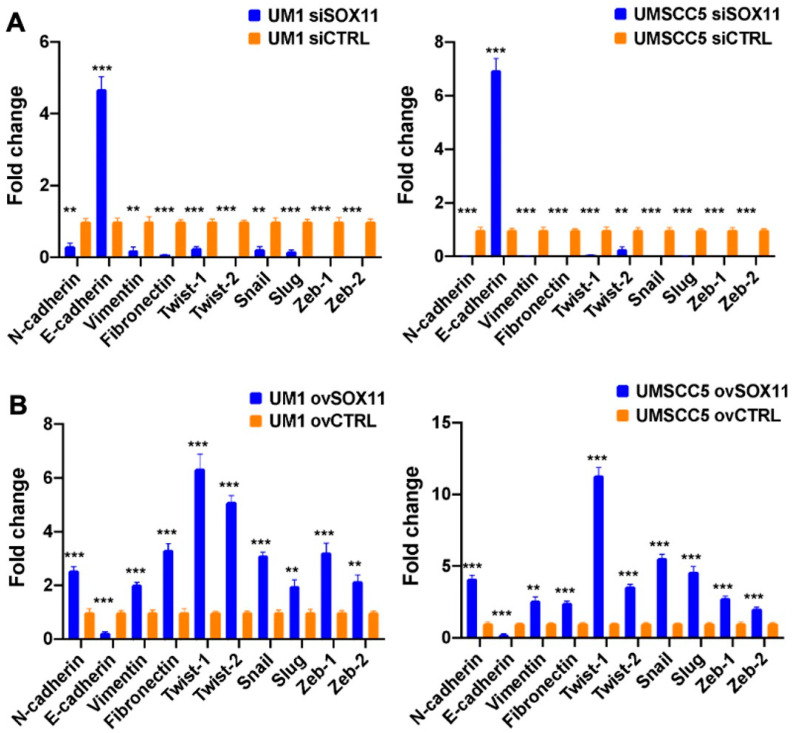
SOX11 upregulates the gene expression of EMT-TFs in UM1 and UMSCC5 cells. (**A**) qPCR analysis of the gene expression of EMT-TFs, including Twist1, Twist2, Snail, Slug, Zeb1, and Zeb2, and N-cadherin, E-cadherin, vimentin, and fibronectin in UM1 and UMSCC5 cells with SOX11 knockdown (siSOX11). (**B**) qPCR analysis of the gene expression of EMT-TFs, including Twist1, Twist2, Snail, Slug, Zeb1, and Zeb2, and N-cadherin, E-cadherin, vimentin, and fibronectin in UM1 and UMSCC5 cells with SOX11 overexpression (ovSOX11) (n = 3 for all assays, **, *p* < 0.01; ***, *p* < 0.001).

To verify if SOX11 regulates EMT-TFs, real-time qPCR was performed to obtain the gene expression of EMT-TFs, including Twist1, Twist2, Snail, Slug, Zeb1, and Zeb2, and N-cadherin, E-cadherin, vimentin, and fibronectin in UM1 and UMSCC5 cells with Sox11 knockdown or overexpression (Figure 4). In UM1 and UMSCC5 cells, the siSox11 knockdown significantly promoted E-cadherin gene expression, whereas the gene expression of N-cadherin, vimentin, fibronectin, and EMT-TFs (Twist1, Twist2, Snail, Slug, Zeb1, and Zeb2) was inhibited by siSox11 knockdown (Figure 4A). On the contrary, overexpression of SOX11 promoted the gene expression of N-cadherin, vimentin, fibronectin, and EMT-TFs (Twist1, Twist2, Snail, Slug, Zeb1, and Zeb2) and inhibited the gene expression of E-cadherin in both UM1 and UMSCC5 cells (Figure 4B).

### 3.5. Correlation Analysis of Sox11 Gene Expression with EMT-TFs, Vimentin, Fibronectin, N-Cadherin, and E-Cadherin Expression in HNSCC Tissues

**Figure 5:**
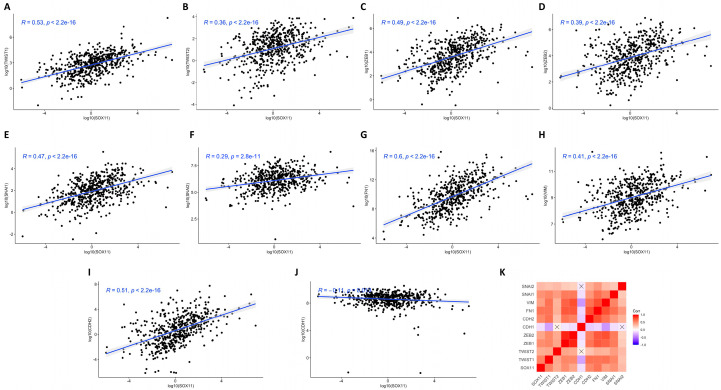
Pearson correlation analysis of the gene expression levels of Sox11 and EMT-TFs in HNSCC tissues based on the TCGA datasets. Data obtained from TCGA were used to graph gene expression correlation between Sox11 and (**A**) Twist1, (**B**) Twist2, (**C**) Zeb1, (**D**) Zeb2, (**E**) Snai1, (**F**) Snai2, (**G**) VIM, (**H**) FN1, (**I**) CDH2, and (**J**) CDH1. (**K**) Heatmap summarizes the findings.

Gene expression data obtained from TCGA were used for correlation analyses between Sox11 and EMT transcription factors, vimentin, fibronectin, N-cadherin, and E-cadherin in HNSCC tissues. Positive correlations were observed between the gene expression of Sox11 and EMT-TFs, including Twist1 (Figure 5A), Twist2 (Figure 5B), Zeb1 (Figure 5C), Zeb2 (Figure 5D), Snai1 (Figure 5E), and Snai2 (Figure 5F) in HNSCC patients’ tissues. Moreover, a positive correlation was observed between the gene expression of Sox11 and vimentin (Figure 5G), fibronectin (Figure 5H), and N-cadherin (Figure 5I), whereas a negative correlation was observed between Sox11 and E-cadherin gene expression levels (Figure 5J). The heatmap summarizes positive correlations between SOX11 and EMT transcription factors (EMT-TFs), including vimentin, fibronectin, and N-cadherin, whereas a negative correlation was observed between SOX11 and E-cadherin (Figure 5K).

### 3.6. SOX11 Regulates the Expression of TWIST1 in HNSCC Cells

**Figure 6:**
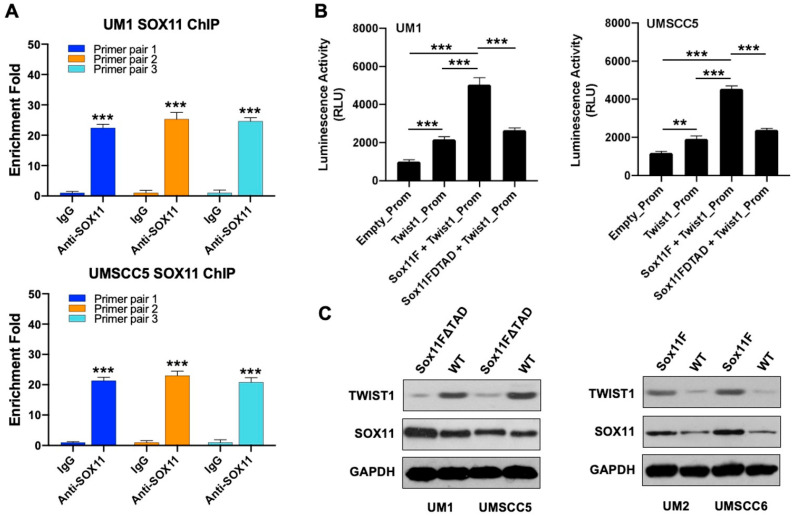
SOX11 upregulates TWIST1 in HNSCC cells. (**A**) ChIP-qPCR analysis of anti-SOX11 immunoprecipitated DNA fragments from UM1 and UMSCC5 cells with 3 pairs of primers for the Twist1 gene promoter. All data were measured in triplicate. ***, *p* < 0.001. (**B**) Luciferase reporter assays of UM1 and UMSCC5 cells transfected with empty promoter reporter vector, Twist1 gene promoter reporter (Twist1-Prom), Twist1-Prom and Sox11F, or Twist1-Prom and Sox11FΔTAD. Data were measured in triplicate. **, *p* < 0.01; ***, *p* < 0.001. (**C**) Western blot analysis of SOX11 and TWIST1 in UM1 and UMSCC5 cells transfected with Sox11FΔTAD (mutant) and in UM2 and UMSCC6 cells transfected with Sox11F. WT: Wild type.

ChIP assays were performed in UM1 and UMSCC5 cells using anti-SOX11 antibodies to determine if SOX11 binds to the Twist1 gene promoter in HNSCC cells. Three primer sets targeting different regions of the Twist1 promoter (primers 1, 2, and 3) were used for qPCR analysis of the immunoprecipitated DNA. The qPCR analysis is carried out by comparing the data from anti-SOX11 immunoprecipitated samples against the negative control (IgG antibody) to calculate the enrichment fold. As shown in Figure 6A, all three promoter regions exhibited substantially higher enrichment in the SOX11 pull-down samples compared with the IgG control in both UM1 and UMSCC5 cells.

Luciferase reporter assays were then carried out using an empty promoter reporter plasmid, Twist1 gene promoter reporter plasmid, Twist1 gene promoter reporter and Sox11 plasmid, and Twist1 gene promoter reporter and mutated Sox11 (Sox11FΔTAD) plasmid in UM1 and UMSCC5 to further confirm that SOX11 regulates the expression of Twist1 in HNSCC cells. Plasmid integrity and successful transfection were verified by restriction digestion and agarose gel electrophoresis. As shown in Figure 6B, co-transfection of Sox11F with the Twist1 promoter reporter markedly increased luciferase activity in UM1 and UMSCC5, whereas the Sox11FΔTAD mutant failed to produce this enhancement.

Western blot analysis of TWIST1 was performed in UM1 and UMSCC5 cells that were transfected with a Sox11 mutant lacking the transactivation domain (Sox11FΔTAD) and in UM2 and UMSCC6 cells that were transfected with the Sox11 plasmid (Sox11F). In highly invasive HNSCC cells (UM1 and UMSCC5), TWIST1 was downregulated when Sox11FΔTAD was transfected (Figure 6C). On the other hand, in low-invasive HNSCC cells (UM2 and UMSCC6), TWIST1 was upregulated when wild-type Sox11F was transfected (Figure 6C).

### 3.7. SOX11 Promotes Tumor Growth In Vivo

**Figure 7:**
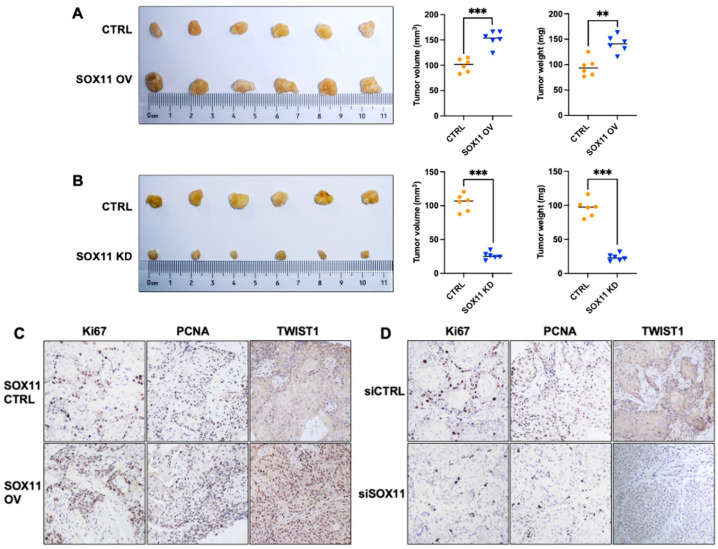
Effect of SOX11 knockdown or overexpression on the growth of xenograft tumors. (**A**) SOX11 overexpression promoted HNSCC tumor growth. (**B**) SOX11 knockdown suppressed HNSCC tumor growth. Both the weight and volume of the xenograft tumors were measured and compared among the SOX11 overexpression, SOX11 knockdown, and corresponding control groups. **, *p* < 0.01; ***, *p* < 0.001. (**C**) IHC analysis of Ki67, PCNA, and TWIST1 expression in xenograft tumor tissues from SOX11 control and overexpression groups. (**D**) IHC analysis of Ki67, PCNA, and TWIST1 expression in xenograft tumor tissues from SOX11 control and knockdown groups.

To evaluate the promoting effect of SOX11 in HNSCC tumor growth, a xenograft mouse model was used with SOX11 knockdown or overexpression. As expected, an increase in the volume and weight of the xenograft tumors was observed in the SOX11 overexpression (SOX11 OV) group when compared with the control group (Figure 7A), whereas the volume and weight of the xenograft tumors were significantly reduced in the SOX11 knockdown (SOX11 KD) group compared to the control group (Figure 7B). The IHC analysis of xenograft tumor tissues showed that the overall staining intensity of PCNA, Ki67, and TWIST1 was markedly increased in the SOX11 overexpression group (Figure 7C) but decreased in the SOX11 knockdown group (Figure 7D) when compared with the control group.

## 4. Discussion

The epidermal growth factor receptor (EGFR), a transmembrane tyrosine kinase receptor, is a key driver of oncogenic signaling. Upon activation by ligands such as EGF, EGFR phosphorylates downstream effectors like STAT3, a transcription factor critical for cancer cell proliferation and survival [36]. While STAT3 activation typically involves phosphorylation by cytokines or growth factors [37], unphosphorylated STAT3 retains partial DNA-binding capacity, suggesting nuanced regulatory roles in transcription [38]. In breast cancer, STAT3 activation via EGFR signaling promotes SOX2 expression [38]. Moreover, recent studies in prostate cancer models indicate that the STAT3-mediated regulation of SOX genes contributes to cancer cell proliferation and survival, suggesting a broader regulatory mechanism conserved across tissues [39]. Aberrant EGFR signaling, particularly nuclear localization and overexpression, is strongly linked to aggressive tumor behavior and poor clinical outcomes in breast, thyroid, and oropharyngeal cancers [40,41,42,43,44]. Emerging evidence positions the EGFR-STAT3 axis as central to tumorigenesis, with combinatorial EGFR/JAK-STAT3 inhibition showing therapeutic potential in cancer diseases [45,46].

Due to the important role of EGFR-STAT3 signaling in tumorigenesis, including HNSCC, we investigated whether SOX11 is upregulated by EGF in four HNSCC cell lines, including two highly invasive UM1 and UMSCC5 and two low-invasive UM2 and UMSCC6. In fact, both UM1 and UM2 cells were derived from the same tumor in a patient with squamous cell carcinoma of the tongue and shared the same TP53 mutation [47]. UM2 cells do not possess stem-like cancer cell properties, whereas a small fraction of UM1 cells do [48]. We compared the expression levels of SOX11 in the EGF-supplemented group and found that EGF upregulates the expression of SOX11 compared to the control group. To further confirm our finding, we inhibited EGF signaling with an EGFR inhibitor and found that the expression of SOX11 was suppressed in HNSCC cell lines. These results indicate that EGF is a potential upstream regulator of SOX11. To investigate if SOX11 is regulated by EGFR-STAT3 signals in HNSCC cells, we performed ChIP assays in HNSCC cells using four different pairs of primers and identified STAT3 as a potential transcription factor that binds to the promoter of Sox11. Furthermore, higher enrichment of DNA fragments was observed in anti-pSTAT3 immunoprecipitated ChIP samples compared to anti-STAT3 immunoprecipitated samples, indicating that pSTAT3 binds to the Sox11 gene promoter more strongly than STAT3. The results from luciferase assays further confirmed that when Sox11 gene promoter constructs and wild-type STAT3 were co-transfected, STAT3 induced significantly higher Sox11 gene promoter activity in HNSCC cells. Our data demonstrate that STAT3 binds to the SOX11 promoter and is sufficient for activating its transcription in HNSCC cells. While we also observed binding of phosphorylated STAT3 (pSTAT3) to the promoter, the functional contribution of pSTAT3 remains unclear. Previous studies have shown that pSTAT3 can exert context-dependent effects on target gene expression, acting as an activator or inhibitor depending on the cell type and signaling context [49]. Therefore, although our luciferase assays specifically reflect the transcriptional activity of STAT3, further studies are needed to elucidate whether pSTAT3 modulates SOX11 expression in HNSCC. In addition, CEMSA experiments demonstrated that STAT3 bound to the predicted consensus DNA sequence of the Sox11 gene promoter based on the JASPAR database. These results collectively indicate that the EGFR-STAT3 signal regulates the expression of SOX11 in HNSCC cells.

EMT-TFs directly or indirectly regulate key epithelial/mesenchymal markers like E-cadherin, N-cadherin, and vimentin during both normal and disease processes [50]. EMT itself can be driven by various mechanisms, including the activation of factors like TWIST, production of ECM-degrading enzymes, altered cell-surface protein expression, and reorganization of the cytoskeleton [51,52]. Crucially, downregulation of E-cadherin promotes increased invasion and a fibroblast-like morphology in epithelial tumor cells from diverse tissues such as breast, lung, bladder, and pancreas [53]. TWIST1 specifically acts as an essential downstream effector of HIF-1α in head and neck cancer, where its knockdown in HIF-1α-overexpressing cells reduces metastatic nodules in vivo [54]. Furthermore, genetic deletion of even one TWIST1 allele blocks the malignant conversion from benign papilloma to invasive carcinoma in a DMBA/TPA skin cancer model [55]. These findings collectively confirm TWIST1′s pivotal role in promoting EMT and tumor invasion.

To investigate if SOX11 regulates the EMT-TFs in HNSCC cells, we compared their gene expression levels in SOX11 knockdown and overexpression groups versus controls. SOX11 knockdown was found to significantly induce E-cadherin gene expression while inhibiting the gene expression of N-cadherin, vimentin, fibronectin, and EMT-TFs, including Twist1, Twist2, Snail, Slug, Zeb1, and Zeb2. On the other hand, SOX11 overexpression significantly inhibited E-cadherin gene expression but induced the gene expression of N-cadherin, vimentin, fibronectin, and EMT-TFs. To further confirm if SOX11 regulates TWIST1, one of the EMT-TFs, we conducted ChIP assays with three pairs of primers and demonstrated that SOX11 bound to the promoter region of the Twist1 gene in HNSCC cells. Because SOX4 and SOX11 share high sequence similarity, it is important to confirm the specificity of the antibody used for the ChIP assay with Western blotting or other immunological methods so as to avoid the cross-reaction of the antibody with other SOXC family members. The luciferase assays confirmed that wild-type SOX11 induced significant promoter activity of the Twist1 gene in HNSCC cells. In addition, overexpression of Sox11FΔTAD, a mutant lacking the transactivation domain, inhibited the expression of TWIST1 in highly invasive HNSCC cells, whereas overexpression of the wild-type SOX11F promoted the expression of TWIST1 in low-invasive HNSCC cells. Although the purpose of our luciferase assay experiments was to demonstrate the requirement of the SOX11 transactivation domain (TAD) for the SOX11-mediated activation of TWIST1, the SOX11-ΔHMG construct may also be included in luciferase assays to further confirm the essential function of the HMG domain in SOX11 and its regulation of TWIST1 expression. Furthermore, SOX11 was found to promote HNSCC tumor growth and the expression of TWIST1 in vivo. These results, collectively, demonstrate that SOX11 regulates the expression of EMT-TFs in HNSCC cells.

While our study delineates the functional role of SOX11 in HNSCC progression, it is worth noting that SOX11 is largely absent in most adult tissues [56,57]. A key future direction is to explore if EGF/STAT3 signaling can ectopically induce SOX11 in non-tumorigenic cells, and if so, whether this prompts TWIST expression and a tumor-like phenotype. Such evidence would provide critical insight into SOX11′s oncogenic capacity and its contribution to EGF-mediated cellular transformation.

## 5. Conclusions

**Figure 8:**
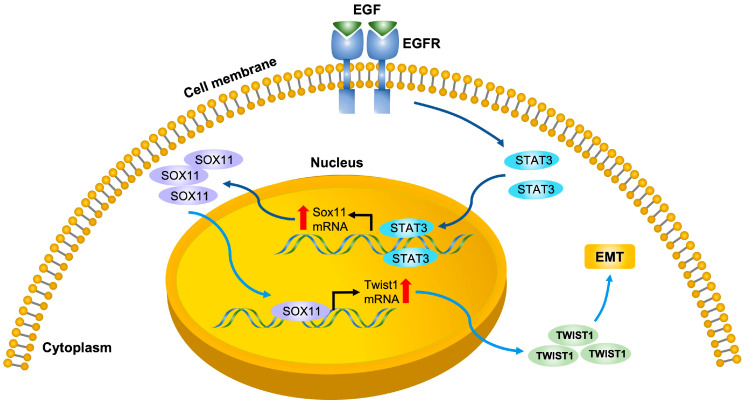
Schematic illustrating that SOX11 is regulated by the EGFR–STAT3 signaling pathway and subsequently activates TWIST1, promoting EMT and HNSCC progression.

Our studies have demonstrated that SOX11 is upregulated by the EGF-EGFR-STAT3 signaling pathway and subsequently activates EMT-TFs, facilitating EMT and tumor progression (Figure 8). These findings highlight the important functional role of SOX11 in HNSCC and provide new insight into the EGFR-driven cancer progression.

## Data Availability

The data used and/or analyzed during the current study are available from the corresponding author upon reasonable request.

## Supplementary Materials

The following supporting information can be downloaded at https://www.mdpi.com/article/10.3390/cells15010084/s1. Table S1: Primers used for qPCR analysis in this study.

## Abbreviations

The following abbreviations are used in this manuscript:

ChIP	Chromatin immunoprecipitation
CEMSA	Capillary electrophoresis mobility shift assay
DMEM	Dulbecco’s modified Eagle’s medium
EGFR	Epidermal growth factor receptor
EGFR-in	EGFR inhibitor
EMT	Epithelial–mesenchymal transition
EMEQA	N-(3-ethynylphenyl)-6,7-bis(2-methoxyethoxy)-4-quinazolinamine
EMT-TFs	EMT transcription factors
HNSCC	Head and neck squamous cell carcinoma
HMG	High-mobility group
IHC	Immunohistochemistry
SRY	Sex-determining region Y
TCGA	The Cancer Genome Atlas
