# Supplementary material for: SOX11 Is Regulated by EGFR-STAT3 and Promotes Epithelial–Mesenchymal Transition in Head and Neck Squamous Cell Carcinoma
Source: Cells. 2026 Jan 4;15(1):84. doi: 10.3390/cells15010084 (PMC12785246; doi:10.3390/cells15010084)
Supplement: Supplementary file 1 [file cells-15-00084-s001.zip › cells-3978577-supplementary.pdf]

Supplementary Table S1. Primers used for qPCR analysis in this study.

Primers	Forward	Reverse
Sox11 primer 1-ChIP	AAAAGGAGAGCAGAGCCCAC	CCTTTGTGCCCAGTCCCC
Sox11 primer 2-ChIP	CCGCCTCTCGAGCTTTAAATTG	CTCACTCCCCGGGTGCAC
Sox11 primer 3-ChIP	CAGAAAAATGCAAAACGGGGG	GGTGCACACACTCGGGCA
Sox11 primer 4-ChIP	CACCGCCTCTCGAGCTTTAA	GCGCCTTTGTGCCCAGTC
Twist primer 1-ChIP	CCGGCGGGGAAGGAAATC	CGAGAGCTGCAGACTTGG
Twist primer 2-ChIP	TGGGCTGCGCTAGGGTTC	CTGCAGACTTGGAGGCTCTT
Twist primer 3-ChIP	GCGGGGAAGGAAATCGCC	TGTCATTGGCCTGACGTGAG
Beta-actin	AGCGAGCATCCCCCAAAGTT	GGGCACGAAGGCTCATCATT
Sox11	CCAGGACAGAACCACCTGAT	CCCCACAAACCACTCAGACT
Twist1	GCCAGGTACATCGACTTCCTCT	TCCATCCTCCAGACCGAGAAGG
Twist2	GCAAGATCCAGACGCTCAAGCT	ACACGGAGAAGGCGTAGCTGAG
Snail	TGCCCTCAAGATGCACATCCGA	GGGACAGGAGAAGGGCTTCTC
Slug	ATCTGCGGCAAGGCGTTTTCCA	GAGCCCTCAGATTTGACCTGTC
Zeb-1	GGCATACACCTACTCAACTACGG	TGGGCGGTGTAGAATCAGAGTC
Zeb-2	AATGCACAGAGTGTGGCAAGGC	CTGCTGATGTGCGAACTGTAGG
CDH1	GCCTCCTGAAAAGAGAGTGGAAG	TGGCAGTGTCTCTCCAAATCCG
CDH2	CCTCCAGAGTTTACTGCCATGAC	GTAGGATCTCCGCCACTGATTC
VIM	AGGCAAAGCAGGAGTCCACTGA	ATCTGGCGTTCCAGGGACTCAT
FN1	ACAACACCGAGGTGACTGAGAC	GGACACAACGATGCTTCCTGAG
